# Allele-specific DNA methylation and gene expression during shoot organogenesis in tissue culture of hybrid poplar

**DOI:** 10.1093/hr/uhae027

**Published:** 2024-01-24

**Authors:** Ying Guo, Yang-Fan Feng, Gang-Gui Yang, Yan Jia, Jie He, Ze-Yu Wu, Hao-Ran Liao, Qi-Xuan Wei, Liang-Jiao Xue

**Affiliations:** State Key Laboratory of Tree Genetics and Breeding, Co-Innovation Center for Sustainable Forestry in Southern China, Key Laboratory of Forest Genetics & Biotechnology of Ministry of Education, Jiangsu Key Laboratory for Poplar Germplasm Enhancement and Variety Improvement, Nanjing Forestry University, Nanjing 210037, China; State Key Laboratory of Tree Genetics and Breeding, Co-Innovation Center for Sustainable Forestry in Southern China, Key Laboratory of Forest Genetics & Biotechnology of Ministry of Education, Jiangsu Key Laboratory for Poplar Germplasm Enhancement and Variety Improvement, Nanjing Forestry University, Nanjing 210037, China; State Key Laboratory of Tree Genetics and Breeding, Co-Innovation Center for Sustainable Forestry in Southern China, Key Laboratory of Forest Genetics & Biotechnology of Ministry of Education, Jiangsu Key Laboratory for Poplar Germplasm Enhancement and Variety Improvement, Nanjing Forestry University, Nanjing 210037, China; State Key Laboratory of Tree Genetics and Breeding, Co-Innovation Center for Sustainable Forestry in Southern China, Key Laboratory of Forest Genetics & Biotechnology of Ministry of Education, Jiangsu Key Laboratory for Poplar Germplasm Enhancement and Variety Improvement, Nanjing Forestry University, Nanjing 210037, China; State Key Laboratory of Tree Genetics and Breeding, Co-Innovation Center for Sustainable Forestry in Southern China, Key Laboratory of Forest Genetics & Biotechnology of Ministry of Education, Jiangsu Key Laboratory for Poplar Germplasm Enhancement and Variety Improvement, Nanjing Forestry University, Nanjing 210037, China; State Key Laboratory of Tree Genetics and Breeding, Co-Innovation Center for Sustainable Forestry in Southern China, Key Laboratory of Forest Genetics & Biotechnology of Ministry of Education, Jiangsu Key Laboratory for Poplar Germplasm Enhancement and Variety Improvement, Nanjing Forestry University, Nanjing 210037, China; State Key Laboratory of Tree Genetics and Breeding, Co-Innovation Center for Sustainable Forestry in Southern China, Key Laboratory of Forest Genetics & Biotechnology of Ministry of Education, Jiangsu Key Laboratory for Poplar Germplasm Enhancement and Variety Improvement, Nanjing Forestry University, Nanjing 210037, China; State Key Laboratory of Tree Genetics and Breeding, Co-Innovation Center for Sustainable Forestry in Southern China, Key Laboratory of Forest Genetics & Biotechnology of Ministry of Education, Jiangsu Key Laboratory for Poplar Germplasm Enhancement and Variety Improvement, Nanjing Forestry University, Nanjing 210037, China; State Key Laboratory of Tree Genetics and Breeding, Co-Innovation Center for Sustainable Forestry in Southern China, Key Laboratory of Forest Genetics & Biotechnology of Ministry of Education, Jiangsu Key Laboratory for Poplar Germplasm Enhancement and Variety Improvement, Nanjing Forestry University, Nanjing 210037, China

## Abstract

Plant tissue regeneration is critical for genetic transformation and genome editing techniques. During the regeneration process, changes in epigenetic modifications accompany the cell fate transition. However, how allele-specific DNA methylation in two haplotypes contributes to the transcriptional dynamics during regeneration remains elusive. Here we applied an inter-species hybrid poplar (*Populus alba* × *P. glandulosa* cv. 84 K) as a system to characterize the DNA methylation landscape during *de novo* shoot organogenesis at allele level. Both direct and indirect shoot organogenesis showed a reduction in genome-wide DNA methylation. At gene level, non-expressed genes were hypermethylated in comparison with expressed genes. Among the genes exhibiting significant correlations between levels of DNA methylation and gene expression, the expression patterns of 75% of genes were negatively correlated with DNA methylation in the CG context, whereas the correlation patterns in the CHH context were the reverse. The allele-biased DNA methylation was consistent during shoot organogenesis, with fewer than one-thousandth of allele-specific methylation regions shifted. Analysis of allele-specific expression revealed that there were only 1909 genes showing phase-dependent allele-biased expression in the regeneration process, among which the allele pairs with greater differences in transcription factor binding sites at promoter regions exhibited greater differences in allele expression. Our results indicated a relatively independent transcriptional regulation in two subgenomes during shoot organogenesis, which was contributed by *cis*-acting genomic and epigenomic variations.

## Introduction

Plant shoot organogenesis is widely employed in tissue culture techniques for genetic transformation and genome editing. The process of organogenesis is based on plant cell totipotency, which is one of the important survival strategies for plants with sessile lifestyles. Plants have significant abilities to drive cells from a differentiated state to a less differentiated state, thereby restoring their identity as pluripotent reprogramming cells. They could re-enter the cell cycle and proliferate to establish new shoot or root apical meristems by plant hormone induction, eventually giving rise to new organs or plantlets [1].

The *de novo* organ regeneration of model organisms has uncovered important molecular crossroads between stress responses and organogenesis, including changes in signal transduction, hormone homeostasis, and the expression of master regulatory transcription factors in the key meristem [2]. In *Arabidopsis* shoot regeneration induced by a two-step protocol, the SCF^TIR1/AFB^ receptor complex acts as auxin receptors to ubiquitinate and degrade aux/IAA transcriptional repressors to release their repression of auxin response factors when incubated on callus-inducing medium (CIM) [3], thereby activating the division of designated pericycle cells. Subsequently, a cytokinin-related phosphorelay consisting of three histidine kinases, five phosphotransfer proteins, and several response regulators was triggered on shoot-inducing medium (SIM), inducing coordinated expression of key transcription factor (TF) coding genes, such as *WUSCHEL* (*WUS*) [4], *SHOOT MERISTEMLESS* (*STM*) [5], and *ENHANCER OF SHOOT REGENERATION 1* (*ESR1*) [6]. In addition, accumulating evidence suggests that epigenetic modifications are the basis for driving cell fate changes and pluripotency establishment, as they can regulate the transcription of a series of reprogramming genes to permit expression to be induced or repressed at the developmental critical periods [7].

DNA methylation is an important and evolutionarily conserved epigenetic mark that controls many vital biological processes in plants. Levels of DNA cytosine methylation vary widely among different plant species, e.g. the genome-wide DNA methylation levels in *Arabidopsis* are 24, 6.7, and 1.7% and those in maize are 86.4, 70.9, and 1.2% for CG, CHG, and CHH contexts, respectively [8]. In research on some horticultural plants, such as Chinese cabbage [9], strawberry [10], and sugar beet [11], it has been found that changes in DNA methylation during regeneration are common and accompanied by diverse patterns of change. In general, DNA hypermethylation at specific loci may hinder regeneration via silencing genes, while whole-genome hypomethylation enhances regeneration through transcriptional activation. For example, DNA methylation in the regulatory regions of *WUS* was lost in the *Arabidopsis* loss-of-function mutant for the DNA methyltransferase *met1*, leading to increased *WUS* expression to improve shoot regeneration [12]. The participation of DNA methylation in gene expression has proved to be highly variable in recent studies [13, 14], while the dynamic effects of phase-dependent DNA methylation on regeneration are not fully understood.

The phenomenon where a hybrid outperforms its parents in terms of growth rate, mature biomass, and reproduction is known as heterosis [15]. In plant breeding, especially tree breeding, elite clones with high heterozygosity were selected to exploit heterosis in growth and/or stress tolerance [16]. Research on cultivated tomato (*Solanum lycopersicon* Mill.) has reported heterosis for adventitious shoot regeneration in terms of maximum shoot multiplication rate [17]. Significant positive heterosis effects were also observed in some crosses of different wheat genotypes for callus induction and plant regeneration [18]. A study has shown that DNA methylation in rice hybrid seeds plays crucial roles in initiating, regulating, and maintaining heterosis of *F*_1_ hybrid plants [19]. Recently, the emergence of high-throughput sequencing technology has provided opportunities to study heterosis at the molecular level, and the accumulation of multi-omics data has expanded scientific understanding of the molecular mechanisms of heterosis. Among them, analysis of allele-specific expression (ASE), ‘a difference in expression levels between alleles due to differences between the allele sequences’ [20], may be an important approach for explaining the classical genetic hypothesis of heterosis: dominance, overdominance, and epistasis [21]. Nevertheless, it is unclear whether and how DNA methylation can drive allele-biased expression to ensure cell fate transition during regeneration, especially in highly heterozygous woody plants.

As an integral part of human life, forests provide long-term benefits to society through provisioning of timber products, food sources, and biomass energy. However, several limitations, such as excessive phenolic exudation, vitrification, and low shoot proliferation, are pronounced in tree tissue culture to inhibit regeneration rate and molecular breeding efficiency [22]. Poplar 84 K (*Populus alba* × *P. glandulosa*) is a fast-growing poplar hybrid, and as a model species it plays an important role in fundamental research in forest molecular biology. In this study we not only focused on changes in epigenetic landscapes during *de novo* shoot organogenesis (DNSO) of hybrid poplar clone 84 K, but also analyzed allelic bias at each regeneration phase. We further emphasize the epigenetic regulatory roles of DNA methylation in gene/allele expression during *in vitro* culture, which would help in the development of strategies to enhance regenerate capability in recalcitrant tree species and provide important clues for the effective multiplication and genetic improvement of trees.

## Results

### DNA methylation landscape of 84 K poplar during shoot regeneration

**Figure 1:**
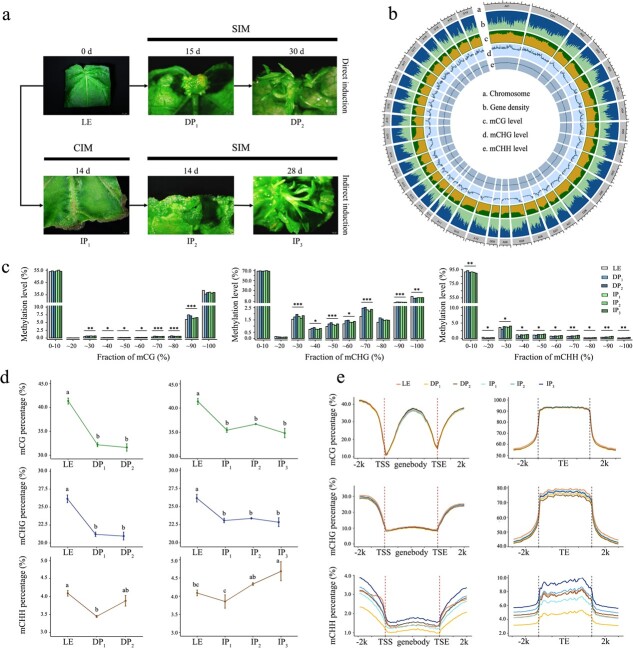
Characterization of the DNA methylome of 84 K poplar during the process of direct and indirect shoot organogenesis. **a** Illustration of samples collected for genome-wide bisulfite sequencing. Leaves from 40-day-old seedlings were used as leaf explant (LE); shoots grown from LEs at 15 DAC (DP_1_) and 30 DAC (DP_2_) on SIM during direct organogenesis, as well as callus or shoot samples at 14 DAC (IP_1_) on CIM and14 DAC (IP_2_), 28 DAC (IP_3_) on SIM during indirect organogenesis were collected. Scale bar = 0.2 cm. **b** Gene density and methylation levels on 19 chromosomes of the two subgenomes of *P. alba* and *P. glandulosa* in LE samples. **c** Distribution ratio of average methylation levels of mCG, mCG, mCHG, and mCHH in samples from different phases of shoot regeneration. *P* values were calculated by one-way ANOVA: *P < 0.05; **P < 0.01; ***P < 0.001. **d** Difference analysis of methylation levels between samples at different phases in the CG, CHG, and CHH contexts. Lower-case letters represent a significant difference between samples (*P* < 0.05). **e** Characterization of the DNA methylation pattern along genes and TEs.

We performed *de novo* shoot organogenesis using leaves from 84 K poplar as explants, inducing them under tissue culture conditions by a one- or two-step method, i.e. direct and indirect induction (Fig. 1a). To reveal dynamic methylation modification features, leaf explant (LE) samples and samples from two and three key biological phases (pluripotency acquisition, IP_1_; shoot progenitor formation, DP_1_ and IP_2_; and shoot outgrowth, DP_2_ and IP_3_) were collected for genome-wide bisulfite sequencing during direct and indirect induction, respectively [23]. Three biological replicates from each stage were sequenced at a mean sequencing depth of 30×, and the biological replicates were highly reproducible, with Pearson correlation coefficient >0.85 (Supplementary Data Table S1).

In the DNA methylation single-base resolution map of leaf explants, ~10.50% of total methylcytosines (mCs) were identified, including 41.37% mCG, 26.16% mCHG, and 4.09% mCHH (Fig. 1b). Approximately 44 and 22% of the mCG and mCHG loci exhibited high methylation rates (>80%), while the rate in mCHH loci was <1% (Fig. 1c). Significant genome-wide hypomethylation of all CG, CHG, and CHH contexts was observed in DP_1_ and IP_1_ samples, whose mCs were decreased respectively by 16.67 and 9.14% compared with the LE (Fig. 1d and Supplementary Data Fig. S1). In subsequent samples, the dynamic trends of mCG and mCHG were similar and relatively stabilized, while the methylation frequencies of cytosines in CHH contexts gradually increased.

At the gene level, average methylation profiles showed gene body methylation with a strong decrease at the gene transcription start sites (TSSs) and the end sites (TESs) in the CG context; relative depletion of DNA methylation percentage in genic regions compared with the surrounding sequences was observed in CHG and CHH contexts (Fig. 1e). The high methylation levels of all contexts were found in transposable element (TE) bodies and were sharply reduced in their surrounding sequences, with short interspersed nuclear elements (SINEs) having the highest level of methylation (Fig. 1e and Supplementary Data Fig. S2). Although all samples in each stage were very similar in the CG context, it could be observed that the mCHG percentage of genes and TE regions gradually decreased during direct and indirect shoot regeneration, as well as the first decrease and then increase of mCHH percentage. The cell fate transitions were accompanied by alterations of the DNA methylation landscape, and we further recognized that diverse reactions of sequence contexts (mCG, mCHG, and mCHH) intricately drove plant regeneration.

### Global dynamic of phase-dependent DNA methylation

**Figure 2:**
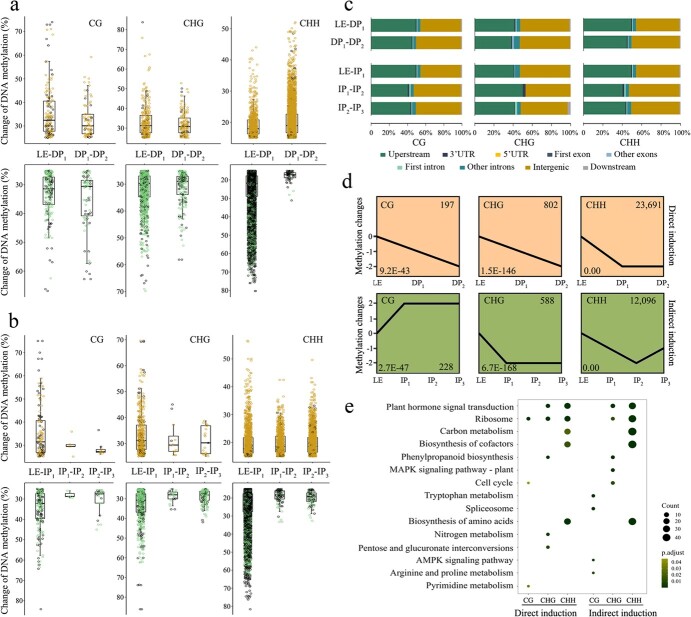
Identification of DMRs between samples from different phases during shoot regeneration. **a**, **b** Methylation changes in each DMR during direct and indirect shoot organogenesis processes, respectively. The upper panel displays DMR with increased methylation, while the lower panel displays DMR with decreased methylation, and among them the black circle indicates that the methylation level of DMRs transitioned to high (>80%) or low (<20%) levels. **c** Relative fraction of DMR distribution in genic regions. **d** Time-resolved analysis of DNA methylation levels of all identified DMRs in CG, CHG, and CHH contexts. The number on the left represents the *P* value of each temporal expression profile, while the number on the right represents the number of DMRs in the profile. **e** Functional analysis of DMGs using the KEGG database.

Explants undergo dedifferentiation and redifferentiation to produce new organs and even the whole plant in response to external stimuli. To characterize DNA methylation differences during shoot regeneration, we successively identified the differentially methylated regions between samples at each DNSO phase. The CHH context encompassed the highest numbers of differentially methylated regions (DMRs, 200-bp bins), especially in the early stages of regeneration, such as 29 274 and 20 849 CHH DMRs and only 1882 and 1282 CG-CHG DMRs in two comparative groups of LE versus DP_1_ and LE versus IP_1_ (Fig. 2a and b). Strikingly, >75% of CHH DMRs were converted from middle or high methylation levels in the LE samples to hypomethylation (<20%) in DP_1_ and IP_1_ samples, which revealed a potential role of early-phase mCHH hypomethylation in exerting pluripotency of differentiated cells. Methylation was relatively stable during the subsequent process of shoot outgrowth, and the number of DMRs significantly decreased in all sequence backgrounds.

The distribution regions of DMRs were relatively consistent in the genome among the five comparative groups, mainly distributed in the intergenic regions and promoter regions (Fig. 2c). For example, an average of 46.26, 41.30, and 37.93% of CG, CHG, and CHH DMRs were distributed on promoters (2-kb upstream region of genes), regulating the expression of genes responsible for reprogramming [24]. All DMRs in three sequence contexts were clustered using the Short Time-series Expression Miner (STEM) algorithm to investigate DNSO-related DNA methylation patterns. Interestingly, the time-course analysis found that the methylation levels of DMRs among direct induction DNSO samples were most significant in linear decreasing patterns in all CG, CHG, and CHH contexts (Fig. 2d). For the six most significant patterns in Fig. 2d, we identified 7666 differentially methylated genes (DMGs), which were defined as genes with one or more DMRs within the gene body or within their 2-kb up- and downstream regions (Supplementary Data Table S2). Functional analysis showed these DMGs were enriched in the regeneration-related pathways of plant hormone signal transduction, ribosome, and carbon metabolism, indicating that loci of key genes involved in the *de novo* shoot organogenesis process were dynamically modified by DNA methylation (Fig. 2e).

### Genome-wide association between DNA methylation and gene expression

**Figure 3:**
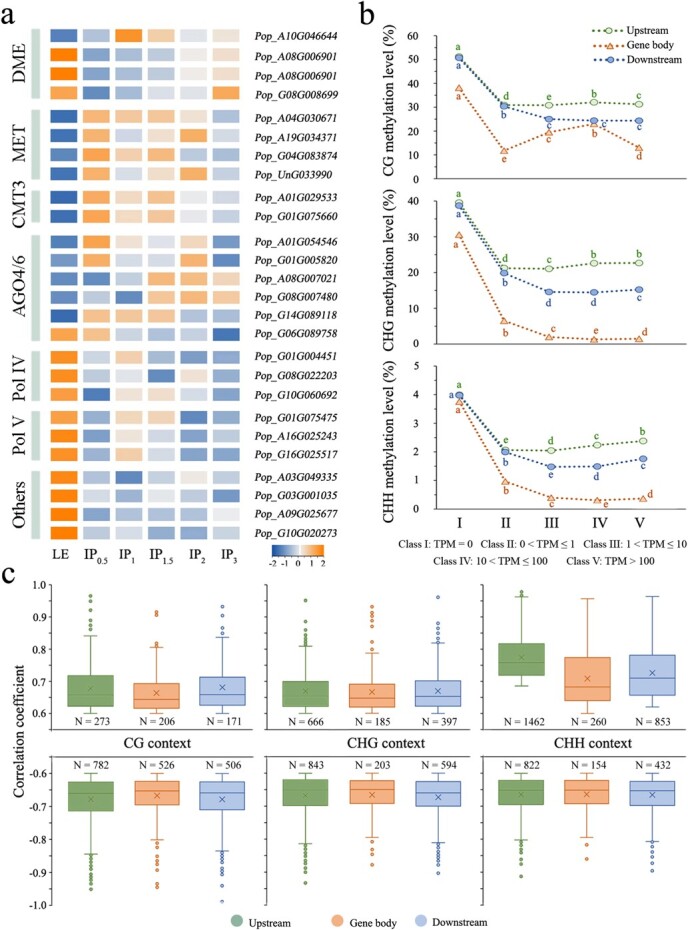
Association between DNA methylation and gene expression. **a** Gene expression patterns related to DNA methylation establishment, maintenance, and demethylation during shoot regeneration of 84 K poplar. Colour scale indicates gene expression levels. **b** Comparison of methylation levels between five classes of genes based on expression levels for gene body and upstream and downstream (flanking 2-kb regions). Small letters represent a significant difference in methylation levels among different groups of genes (*P* < 0.05). **c** Pearson correlation between gene expression and methylation levels of 80 bins located in gene body and upstream and downstream regions (|*r*| > 0.6 and *P* < 0.05, significant correlation). The number of genes whose methylation level was closely related to the level of their expression was counted in CG, CHG, and CHH contexts.

In order to explore the role of DNA methylation in transcriptional regulation of reprogramming genes to ensure they were induced or repressed at the correct developmental window of time, we performed genome-wide association analysis between gene expression and DNA methylation. During the process of indirect shoot organogenesis via callus, samples cultured on CIM and SIM for 7 days (IP_0.5_ and IP_1.5_) were additionally collected for RNA-seq to gain a more comprehensive understanding of transcriptional dynamics. We first focused on the dynamic expression changes of 155 methylation pathway-related genes during DNSO, which are involved in the establishment and maintenance of DNA methylation as well as active demethylation. Compared with LE, the expression of multiple genes encoding DNA methyltransferases (*MET* and *CMT3*) was significantly increased by >5-fold in IP_0.5_ samples, while the expression of several DNA demethylase genes (*DME*s) was downregulated >2-fold (Supplementary Data Table S3 and Fig. 3a). Further, we identified a lot of differentially expressed genes (DEGs) on the canonical RNA-directed DNA methylation (RdDM) pathway. Consistent with the overall decrease in DNA methylation, a significant decrease in the expression of Pol V-encoded genes that specifically recruit downstream effectors to chromatin to catalyze *de novo* DNA methylation was observed in IP_s_ samples (Supplementary Data Table S3 and Fig. 3a).

Next, we divided genes into five classes based on their expression levels in different DNSO phase samples. Each gene and its up- and downstream regions were divided into 80 bins, and the methylation rate of bins was calculated to reveal the effect of methylation levels in different genomic regions on gene expression. We observed a consistent trend in the gene body and its upstream and downstream regions, where genes with TPM (transcripts per kilobase of exon model per million mapped reads) = 0 in Class I showed higher levels of methylation in all sequence contexts (Fig. 3b). For Class II–V genes, the methylation levels in the upstream region of the genes roughly increased with increasing gene expression, while changes in their downstream region followed the opposite pattern. Because the distribution patterns of gene body methylation were different among CG, CHG, and CHH sequence contexts (Fig. 1e), there were differences in the relationship between methylation and gene expression levels in the three contexts. Genes that were highly and lowly expressed had opposite levels of mCG at the gene body, while genes in Class II to Class IV had significantly increased average mCG levels (Fig. 3b).

Additionally, Pearson correlation analysis was conducted to define the relationship between the methylation rate of each bin and the expression of corresponding genes. In CG, CHG, and CHH sequence contexts, 2562, 3050, and 5526 bins closely related to gene expression (|*r*| > 0.6, *P* < 0.05) were identified from ~7 million bins, which were located in 2464, 2879, and 3983 genes, respectively (Fig. 3c and Supplementary Data Table S4). Focusing on a number of regeneration-related genes involved in biological process of transcriptional regulation, epigenetic regulation, and hormone synthesis and transduction [1], we found that the dynamic transcript levels of *YUC4*, *PIN1*, and *SCR* were negatively correlated with methylated regions located in the promoter or gene body, whereas the expression of *ARF3*, *PAT1*, and *PLT5* was positively correlated with them. Interestingly, it was also found that the expression patterns of 75% of genes were negatively correlated with DNA methylation in the CG context, whereas the correlation patterns in the CHH context were the reverse, indicating that the roles of DNA methylation in gene regulation are diverse and go beyond simple gene repression. In addition, we found that >60% of genes closely related to methylation were DEGs, while the remaining non-DEGs were mainly involved in pathways of mRNA surveillance, nucleocytoplasmic transport, and spliceosome (Supplementary Data Table S5).

### Allele-specific expression analysis of samples from each shoot regeneration phase

**Figure 4:**
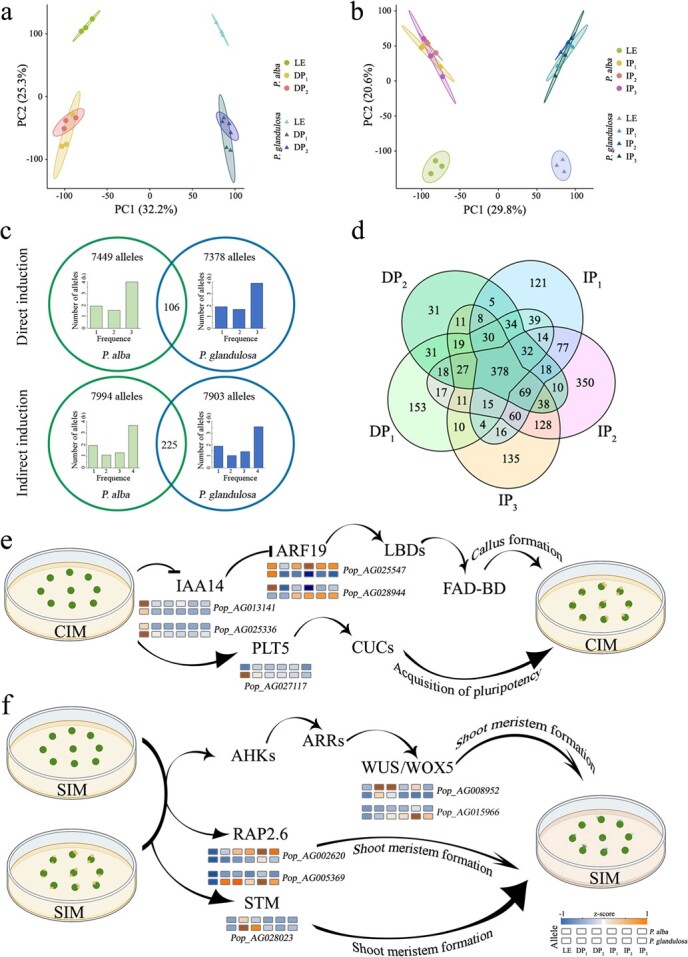
Allele-specific expression (ASE) during shoot regeneration. **a**, **b** PCA score plots of allele expression in samples from each phase of direct and indirect shoot organogenesis, each point representing an independent biological replicate. **c** Venn diagram displaying the numbers of ASEGs and the frequency statistics of their occurrence in each phase sample. **d** Venn diagrams displaying the numbers of phase-dependent ASEGs in samples at regenerate phases compared with the explant samples. **e**, **f** Allelic expression patterns of universal master regulators of callus formation and shoot regeneration, respectively. Colour scale indicates gene expression levels.

Tissue culture technology has been successfully applied in the model tree species of poplar, yet its heterosis and epigenetic regulation remains unclear, which limits our understanding of the regeneration mechanism of highly heterozygous woody plants. To explore the regulation of allelic parental bias and DNA methylation during shoot regeneration, we firstly identified 29 294 allele pairs between *P. alba* (maternal) and *P. glandulosa* (paternal) subgenomes using JCVI, BLAST, and OrthoFinder software (Supplementary Data Table S6). The principal component analysis (PCA) score plot of allelic expression showed that the maternal and paternal alleles in the samples from each phase of direct and indirect shoot organogenesis were significantly separated along the first axis (Fig. 4a and b). ASE analysis was used to measure the expression levels of alleles, which revealed that a total of 8408 and 8374 maternal and paternal bias alleles, referred to as allele-specific expressed genes (ASEGs), were identified in samples from all phases of direct and indirect shoot organogenesis (Supplementary Data Table S7). About 50% of ASEGs showed consistent strong parental-biased expression across all phases, whereas only 106 and 225 ASEGs were found to exhibit different parental bias at different phases during direct and indirect shoot organogenesis (Fig. 4c). To further explore how allele-specific expression responds to dynamic developmental processes, we analyzed phase-dependent ASEGs in different phase samples compared with the explant samples. A total of 1909 phase-dependent ASEGs were identified in samples at all DNSO phases using the DESeq2 package nested multi-factor design (Supplementary Data Table S8). The 378 phase-dependent ASEGs showed consistent biased expression in all samples and 31–350 phase-dependent ASEGs were also discovered in five phases of the samples (Fig. 4d). In addition, we found multiple phase-dependent ASEGs that encode TFs involved in the process of callus formation [1], such as *IAA14*, *ARF19*, and *PLT5* (Fig. 4e). Compared with the explant samples, the biased expression of *ARF19* (*Pop_AG028944*) was reversed in subsequent phase samples during shoot regeneration. Further, a few ASEGs encoding TFs involved in shoot regeneration were identified [1], such as *RAP2.6*, *STM*, *WUS*, and *WOX5* (Fig. 4f). For example, *WOX5* (*Pop_AG015966*), which activates cytokinin signaling and positively triggers shoot regeneration, exhibited paternal-bias expression with 5-fold upregulation during the DNSO, which may be an important target for improving regeneration ability.

### Role of *cis* and *trans* effects in allele-biased expression

**Figure 5:**
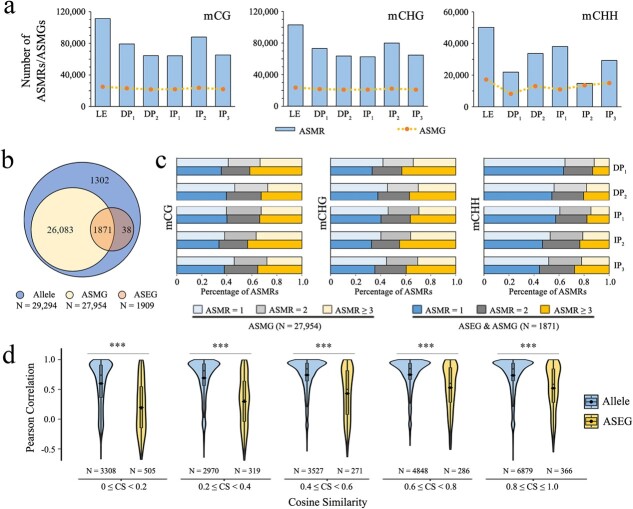
*Cis*-regulatory analysis of ASE. **a** Numbers of ASMRs and ASMGs in CG, CHG, and CHH contexts. **b** Venn diagram displaying the numbers of identified alleles, ASMGs, and phase-dependent ASEGs. **c** Statistical analysis was conducted on the percentage of 27 954 ASMGs and 1871 phase-dependent ASEGs containing different numbers of ASMRs. **d** Correlation analysis between TFBSs cosine similarity of alleles or phase-dependent ASEGs and their expression levels in five groups. Asterisks represent a significant difference in correlation coefficient of each group between alleles and phase-dependent ASEGs (***P < 0.001).

We appraised allele-specific DNA methylation regions (ASMRs) in samples at each DNSO phase using the methylKit package, which were considered as allele-biased DNA methylation regions that contain DMRs between a pair of alleles. The most ASMRs were identified in LE samples, while the number of ASMRs decreased in DP_1_ and IP_1_ samples due to a phased decrease in overall DNA methylation (Fig. 5a). Notably, the methylation bias of less than one-thousandth of ASMRs shifted during the regeneration process in all sequence contexts (Supplementary Data Fig. S3). Alleles containing one or more ASMRs were defined as allele-specific DNA methylation genes (ASMGs); 89.61, 84.71, and 63.01% of all alleles were identified as ASMGs in the CG, CHG, and CHH contexts, respectively (Fig. 5a and Supplementary Data Table S9). The number of ASMGs remained relatively stable in the samples of each phase in the CG and CHG contexts, and it showed a trend of first decreasing and then increasing in the CHH context. To investigate whether DNA methylation variations are associated with allele-specific expression, 1871 of 1909 were also identified as ASMGs among the identified phase-dependent ASEGs (Fig. 5b). About 40% of the phase-dependent ASEGs contained only one ASMR in each sequence context, and the 1781 phase-dependent ASEGs had a little higher proportion of alleles that contained three or more ASMRs than the remaining 26 083 ASMGs (Fig. 5c), while significant differences were only observed in the CHG context (*P* < 0.05, Supplementary Data Table S10). Not as expected, significantly more ASMRs were not found in these phase-dependent ASEGs, indicating that DNA methylation alone might not play a predominant role in driving ASE.

Because the allele-biased expression phenomenon of preferentially expressing a particular allele under regulatory factors can be attributed to both genetic and epigenetic variations [25], we next dissected the causal relationships between allele-biased expression and variants in *cis*-regulatory elements (transcription factor binding sites, TFBSs). Firstly, we searched for 2 160 988 TFBSs in 2000-bp upstream promoters of all alleles using the MEME web-based tool. The similarity of two allelic promoters was then measured by the cosine similarity of their corresponding TFBSs (see Materials and methods section). Compared with other alleles, the cosine similarity of most phase-dependent ASEGs’ TFBSs was lower, indicating more differences in TFBSs at their promoters (Fig. 5d). We found a significant association between TFBS differences in phase-dependent ASEGs and allele expression (*P* < 0.01), meaning that the greater the TFBS difference between a pair of alleles, the lower the correlation between allelic expression (Fig. 5d). It is also worth noting that nearly all identified TFBSs showed no or extremely low levels of DNA methylation, which might not affect the affinity of TFs for their binding sites. These findings suggest that genetic variations of *cis*-regulatory elements play an important role in modulating allele-biased expression of 84 K poplar during shoot regeneration.

## Discussion

Forest trees are an important component of biodiversity as well as a renewable source of food, fuel, and wood products. However, the rapid regeneration of most tree species is limited with several inherent bottlenecks because trees are generally slow-growing, have a long lifespan, are sexually self-incompatible, and have high heterozygosity, especially in the context of changing climate. DNA methylation is closely associated with cell fate transition, which has been confirmed in the model plant *Arabidopsis* and several horticultural crops. To date, it is unclear whether and how DNA methylation is involved in the *de novo* organogenesis of woody plants during *in vitro* tissue culture. Poplars are considered one of the primary systems for studying many aspects of tree biology and the preferred candidates for implementation of intensive, biotechnology-driven forestry [26]. In this work, we systematically characterized the global dynamics of DNA methylation during the DNSO of hybrid poplar clone 84 K, and discussed the possible regulation of DNA methylation in gene/allele expression, which will contribute to understanding of the role of epigenetic regulation in tree regeneration.

### Shoot organogenesis experienced a reduction in genome-wide DNA methylation

Two basic strategies used for DNSO of poplar are direct regeneration and indirect regeneration via an intermediate callus phase. In all contexts of CG, CHG, and CHH, genome-wide hypomethylation of 84 K poplar was predominantly observed at the initial phase of both direct and indirect induction (Fig. 1d). Although hypomethylation is predominantly observed, quite a few variations of methylation patterns during callus formation are recognized among species. The induction of hypermethylation has been reported; for instance, mCHG increases during callus formation from leaf explants in *Fragaria vesca* [10] and *Arabidopsis* [27]. Interestingly, we noticed the phase variation of mCHH, such as more than two-thirds of CHH DMRs were converted to hypomethylation (<20%) during the LE-to-DP_1_/IP_1_ phase transition (Fig. 2a and b). The CHH hypomethylation may contribute to balancing genome stability and activating the expression of pluripotency-related genes [28]. Similarly, the critical initiation of stage-specific mCHH hypomethylation of cotton somatic embryogenesis marked and distinguished embryonic redifferentiation. RdDM is the main molecular pathway responsible for *de novo* DNA methylation in all contexts and maintains asymmetric CHH methylation [29]. Through the RdDM pathway, DRM2 maintains CHH methylation at RdDM target regions. Further, CHH methylation is also affected by MET1, since MET1-dependent methylation can be recognized by SUVH2 and SUVH9 for recruitment of POL V at some RdDM loci [30]. DEG analysis showed that the expression of multiple genes involved in the RdDM pathway decreased significantly, such as *NRPE1* and *SUVH9*, which might be responsible for the CHH hypomethylation at pluripotency acquisition phase (Fig. 3a and Supplementary Data Table S3).

### Significant correlations between levels of DNA methylation and gene expression

An integrated live imaging and single-cell transcriptomics study showed that the isolated mesophyll protoplast induced enhanced expression variation at the genome level [31]. Alterations in epigenetic modifications are expected to further increase the ‘scrambling’ of gene expression to promote regeneration. There are reports that DNA methylation participates in the dedifferentiation and differentiation process of somatic cells by regulating gene expression [32]. Consistent with previous studies, changes in methylation state were observed prominently in promoter regions during the DNSO process (Fig. 2c). We noticed that multiple genes involved in the plant hormone signal transduction pathway contained DMRs, with regional methylation levels significantly decreasing over culture time (Fig. 2e). Meanwhile, we found the methylation levels of non-expressed genes were significantly higher than those of transcriptionally expressed genes (Fig. 3b). In general, hypomethylation is associated with gene activation, while hypermethylation leads to gene silencing. Nevertheless, a few studies have indicated that DNA methylation in the gene body can also be positively correlated to transcription of the gene [33]. Consistently, a positive correlation between the methylation levels of bins located at the gene body/upstream/downstream regions and gene expression were also observed in the CG, CHG, and CHH contexts (Fig. 3c). The methylation regions positively correlated with gene expression may be functional, and were mostly located on genes that negatively regulate transcription and metabolism [34]. For example, we only identified multiple positively correlated methylation regions on the promoter and gene body of *IAA14*, which is an aux/IAA repressor of auxin signaling (Supplementary Data Table S4). We found that the methylation-related non-DEGs were enriched in the mRNA surveillance pathway (Supplementary Data Table S5), which ensures the quality of mRNAs and detects translational errors and degrades abnormal mRNAs through DNA methylation modification [35]. In addition, the way DNA methylation participates in gene regulation is considered highly variable, such as marking alternative intra-genic promoters [36], being affected by TFs at enhancers [37], or itself affecting the binding of TFs [38]. We noted that a few universal master regulators of shoot regeneration, such as *SCR*, *PTL5*, and *ARF3*, contained multiple methylated regions that are closely related to their expression. As discussed above, DNA methylation as a whole emerges both as marker and determinant of cellular identity, affecting the expression of genes involved in shoot regeneration of 84 K poplar through various molecular pathways.

### *Cis*-acting genomic and epigenomic variations drive allele-biased expression

Since allele-biased expression may lead to phenotypic variation, ASE is proven to be a reliable mechanism for causing heterosis, which has been confirmed in plants such as wheat [18] and rice [21]. Similar to regulation of gene expression, allele-biased expression is affected by genetic and epigenetic variations in response to developmental and environmental change. Our data indicated that 16 782 ASEGs were identified among a total of 29 294 alleles, with >50% of ASEGs exhibiting consistent parent-biased expression in samples at each phase of shoot regeneration (Fig. 4c). Compared with the LEs, 398 out of 1909 phase-dependent ASEGs showed consistently biased expression during shoot regeneration (Fig. 4d). The strong and consistent expression bias is likely caused by the function differences of the parental alleles, which will lead to partial to complete dominance effects on traits regulated by genes. It cannot be ignored that a very few genes showed direction-shifting patterns of allele-biased expression during the DNSO process (Supplementary Data Tables S7 and S8). This discovery supports the previous hypothesis that when both alleles are functional, one allele may function better in some developmental stages and/or environmental conditions, while the other allele may be superior in other circumstances [39]. The dominance and overdominance genetic effects between pairs of alleles may provide an important cause of heterosis in highly heterozygous woody trees. Therefore, the ASEGs identified in our study, such as *RAP2.6*, *STM*, *WUS*, and *WOX5* (Fig. 4f), provide candidates for future heterosis research related to genetic and molecular mechanisms.

The dominant/overdominant effects between *cis-* and *trans*-acting regulation of ASEGs have been widely discussed in research on animals and plants. We dissected the causal relationships between variants in *cis*-regulatory elements and allele-biased expression, showing a significant association between TFBS differences in phase-dependent ASEGs and their expression (Fig. 5e). In addition, we found that the methylation of most TFBSs were at extremely low levels that may not prevent TF binding from triggering allele-biased expression, suggesting that the differential expression of alleles during shoot regeneration is primarily regulated by sequence variation of *cis*-regulatory elements. Due to the inherent stochasticity of gene expression across cells, this means that for each allele some cells may be in a transcription ‘on’ state, while others may be in an ‘off’ state. Similarly, the systematic analysis of ASE at single-cell resolution also revealed that *cis* control in gene expression overwhelmingly manifests as differences in transcriptional burst frequency during embryo development [40]. Here, the allelic-specific DNA methylation analysis revealed the presence of ASMR between almost all alleles, but there was no significant difference in the number of ASMRs between ASEGs and other alleles (Fig. 5c). Consistent with our inference, a study on three different cell types of the GenCord human population cohort found that DNA methylation alone could not significantly drive allele-biased expression [33].

Collectively, we revealed diverse patterns of DNA methylation and ASE in response to developmental cues during the *de novo* shoot organogenesis process, providing important clues for the effective reproduction of forest trees. Recent technological innovations have made possible the better characterization of DNA methylation and allele expression across individual cells to explore the molecular basis behind diverse modes of regeneration. With the continuous emergence of epigenetic regulatory information, locus-specific modification of DNA methylation status through advanced gene editing tools will facilitate precisely targeted improvement of regeneration efficiency for recalcitrant high-value trees.

## Materials and methods

### Plant materials and growth conditions

According to the previous method [41], after modifications, we adopted one-step direct and two-step indirect methods for shoot regeneration of poplar 84 K. Here, the third to fifth leaves from 40-day-old tissue culture seedlings of diploid poplar 84 K were used as leaf explants (LEs) for *de novo* shoot organogenesis (DNSO). For direct organogenesis, the 1 × 1 cm^2^ LEs were cultured directly on shoot-inducing medium (SIM), which was composed of 0.05 mg/l NAA, 0.5 mg/l 6-BA, 0.8% agar, and 3% sucrose added to MS basic medium; for indirect shoot organogenesis via callus, LEs were grown on callus-inducing medium (CIM), which was composed of 3.2 mg/l NAA, 0.88 mg/l 6-BA, 0.8% agar, and 3% sucrose added to MS basic medium; after incubation for 14 days, calluses were transferred onto SIM (2.22 mg/l 6-BA; other conditions were consistent with those described above the) for 4 weeks of growth under 23 ± 2°C and a long photoperiod (16 h light and 8 h dark). Key phases of poplar DNSO were distinguished as described for *Arabidopsis* [23], including pluripotency acquisition, shoot progenitor formation, and shoot outgrowth. Samples (>1.0 g) from different DNSO phases were collected for transcriptome sequencing and genome-wide bisulfite sequencing. For samples from each phase, three biological replicates were collected from 30 LEs.

### Transcriptome sequencing and data analysis

Samples of LEs and from leaf explants at 15 days after culture (DAC, DP_1_) and 30 DAC (DP_2_) on SIM during direct organogenesis, as well as samples at 7 DAC (IP_0.5_), 14 DAC (IP_1_) on CIM, and 7 DAC (IP_1.5_), 14 DAC (IP_2_), and 28 DAC (IP_3_) on SIM during indirect organogenesis, were subjected to RNA-seq analysis. The samples of DP_1_, DP_2_, IP_1.5_, IP_2_, and IP_3_ were regenerated shoots, while the samples of IP_0.5_ and IP_1_ were callus growing along the cut edges. According to the manufacturer’s protocol, total RNA extraction was performed with the Trizol reagent kit (Invitrogen, Carlsbad, CA, USA) to construct the RNA sequencing library. The quality and concentration of total RNAs were examined using a NanoPhotometer^®^ (Implen, CA, USA) and an RNA 6000 Nano Kit (Agilent Technologies, CA, USA), respectively. After further purification of RNA fragments, they were enriched by PCR amplification to construct cDNA libraries and then sequenced on the NovaSeq™ 6000 platform. Paired-end reads of 150 bases were obtained for each sequenced fragment.

To obtain high-quality data, original sequencing reads were filtered using in-house Perl scripts, and then HISAT2 (version 2.1.0) [42] was used to map them onto the reference genome of 84 K poplar [43] (https://db.cngb.org/search/project/CNP0000339/), which contained two subgenomes, *P. alba* and *P. glandulosa*, with 19 chromosomes each. For each sample, read counts for each gene were made by HTSeq (version 0.6.0) [44]. Transcripts per kilobase of exon model per million mapped reads (TPM) were calculated to quantify gene expression abundance and variation. The identification of DEGs (|log_2_FoldChange| > 1 and FDR < 0.05) were performed with R package DESeq2 (version 1.6.3) [45].

### Identification and expression analysis of alleles

A synteny-based strategy and a homology-based method were used to identify alleles of 84 K poplar. To search the genes in the syntenic region, the JCVI (MCScan) package was utilized for performing synteny analysis between the two subgenomes of *P. alba* and *P. glandulosa* with parameter -0.99, and the alleles were considered to be the uniquely paired genes in each syntenic block [46]. For the genes within non-syntenic regions, BLAST software (v2.12.0) was used to perform the bidirectional-best-hits (BBH) method to identify the gene pairs of similar protein sequences in two subgenomes that are more similar to each other than any other gene in the other genome [47, 48]. OrthoFinder software (v2.5.4) was used to search single-copy orthologous genes in the remaining genes [49]. Gene pairs that are best matches and single-copy orthologs to each other are also considered alleles. Subsequently, alleles of 84 K poplar were annotated in customized python script by combining three methods.

Allele-specific expression analysis calculated the differential expression levels of alleles by reading counts from RNA-seq data. A negative binomial generalized linear model implemented in the DESeq2 package was used to identify maternal and paternal bias alleles (genetic-based ASEGs) for each sample. In addition, the DESeq2 package nested multi-factor design was used to identify phase-dependent ASEGs during DNSO, with LE samples as controls. In the analysis, the trends of expression bias between two alleles in LE samples were compared with the trends of bias in samples at other phases. Phase-dependent ASEGs were considered to be gene loci exhibiting differential expressional bias between two alleles at two phases. In the statistic test, the phase effect was considered to be the main factor, the genetic effect was the secondary factor, and their interaction effect was tested for significance. The gene pairs with |log_2_FoldChange| > 1 and FDR < 0.05 were considered as showing allele-biased expression.

### Bisulfite sequencing and methylation analysis

The sequencing libraries of LE, DP_1_, DP_2_, IP_1_, IP_2_, and IP_3_ samples were constructed with the EZ DNA Methylation-Gold™ Kit (D5006). The insert size and effective concentration of libraries were assessed using the Agilent 2100 Bioanalyzer and StepOnePlus™ Real-Time PCR system. The libraries were sequenced at 150 bp paired end by the HiSeq X10 platform. The low-quality reads and adapter sequences were filtered by Trimmomatic (version 0.39) [50] with default parameters. Then, filtered reads were aligned to the reference genome of 84 K poplar (https://db.cngb.org/search/project/CNP0000339/) using Bismark (version 0.22.3) [51] with bowtie2 as the aligner, followed by the removal of PCR duplicates using deduplicate_bismark, and bismark_methylation_extractor was used to call methylation values.

Methylation ratios of cytosines (each cytosine covered at least five times) were calculated as the number of Cs divided by Cs plus Ts. In the CG, CHG, and CHH contexts, DNA methylation levels were estimated by the ratio of methylated cytosines to all cytosines. To calculate the methylation level of TEs and genes, its coordinate was extracted and divided into 80 bins using CGmapTools (version 0.1.2) [52], including 40 bins in the regions encompassing 2 kb upstream and downstream and 40 bins in each body region. Next, the weighted methylation level of each bin was calculated using a python script for all genes or TEs.

### Identification of differentially methylated regions and allele-specific DNA methylation regions

To call the DMRs among samples at different phases, the genome was divided into bins with a length of 200 bp and sliding step size of 100 bp, which were used to detect DMRs by methylKit (version 1.24.0) [53] using default parameters. Only bins that contained no fewer than five cytosines in all samples were selected for DMR analysis, and the number of methylated and unmethylated cytosines in each bins was counted. As reported previously [54], the bins with an absolute methylation difference greater than the thresholds of 0.25, 0.25, and 0.15 for CG, CHG, and CHH and Q-value significance level <0.05 were considered as DMRs. Genes containing one or more DMRs in the gene body and its upstream and downstream 2-kb regions were defined as differentially methylated genes (DMGs). Short Time-series Expression Miner (STEM) [55] was used for cluster analysis of DMRs, and DMGs in the most significantly enriched group shown by the STEM analysis results were used for KEGG enrichment analysis by clusterProfiler [56].

Further, we appraised allele-specific/allele-biased DNA methylation regions (ASMRs) for samples at each DNSO phase. Similarly, the 80 bins from each gene were used to identify DMRs between a pair of alleles by the calculateDiffMeth function in methylKit, which were known as ASMRs. Then, alleles contained one or more ASMRs were defined as allele-specific DNA methylation genes (ASMGs).

### Bioinformatics and statistical analysis

Based on gene expression levels, all genes were divided into five classes (Class I, TPM = 0; Class II, 0 < TPM ≤ 1; Class III, 1 < TPM ≤ 10; Class IV, 10 < TPM ≤ 100; Class V, TPM > 100). Significant differences in methylation levels among the five groups of genes were determined using the Wilcoxon–Mann–Whitney test, with threshold *P* value <0.05. The Pearson correlation between gene expression (TPM) and methylation levels of 80 bins in each gene across LE, DP_1_, DP_2_, IP_1_, IP_2_, and IP_3_ samples was analyzed using cor.test (|*r*| > 0.6 and *P* < 0.05, significant correlation).

For identification of *cis*-regulatory elements, upstream 2-kb sequences of each allele (promoters) were extracted from the 84 K poplar reference genome sequence using the python script and a set of TF binding motifs of *Populus trichocarpa* was downloaded from PlantTFDB [57]. Next, we identified all potential TF binding motifs across interest sequences by comparing them with known motifs using FIMO [58] with the default parameters. As in a previous study, we represented each allele as a vector, where each entry corresponded to a motif in L, with its value equal to 1 if it was predicted to be predicted in the allele, or 0 otherwise [59]. To measure the similarity of the promoters between a pair of alleles, we compared all TFBSs on the promoters by the cosine similarity (CS) of the vectors of the alleles. We divided the alleles into five classes based on their cosine similarity (Class I, 0 ≤ CS < 0.2; Class II, 0.2 ≤ CS < 0.4; Class III, 0.4 ≤ CS < 0.6; Class IV, 0.6 ≤ CS < 0.8; Class V, 0.8 ≤ CS ≤ 1.0), and analyzed the Pearson correlation of gene expression (TPM) between each pair of alleles.

## Supplementary Material

Web_Material_uhae027
