# Supplementary material for: Allele-specific DNA methylation and gene expression during shoot organogenesis in tissue culture of hybrid poplar
Source: Hortic Res. 2024 Jan 24;11(3):uhae027. doi: 10.1093/hr/uhae027 (PMC10967691; doi:10.1093/hr/uhae027)
Supplement: Web_Material_uhae027 [file web_material_uhae027.zip › Figure S.docx]

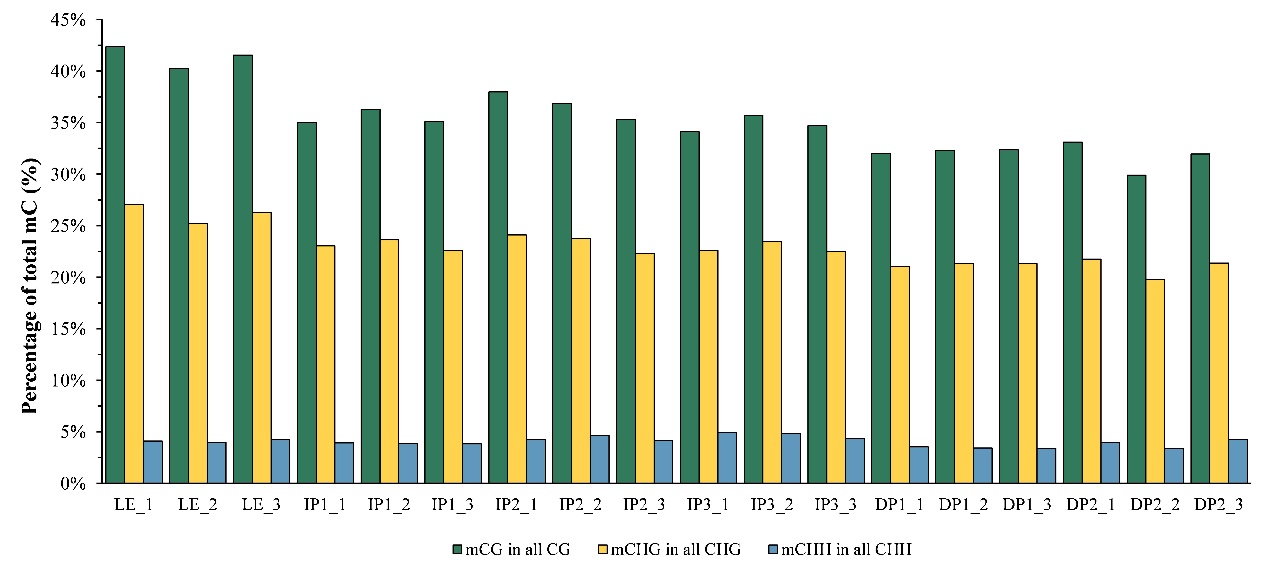


**Figure S1.** Percentages of mCG, mCHG and mCHH in samples from different phases of shoot regeneration.


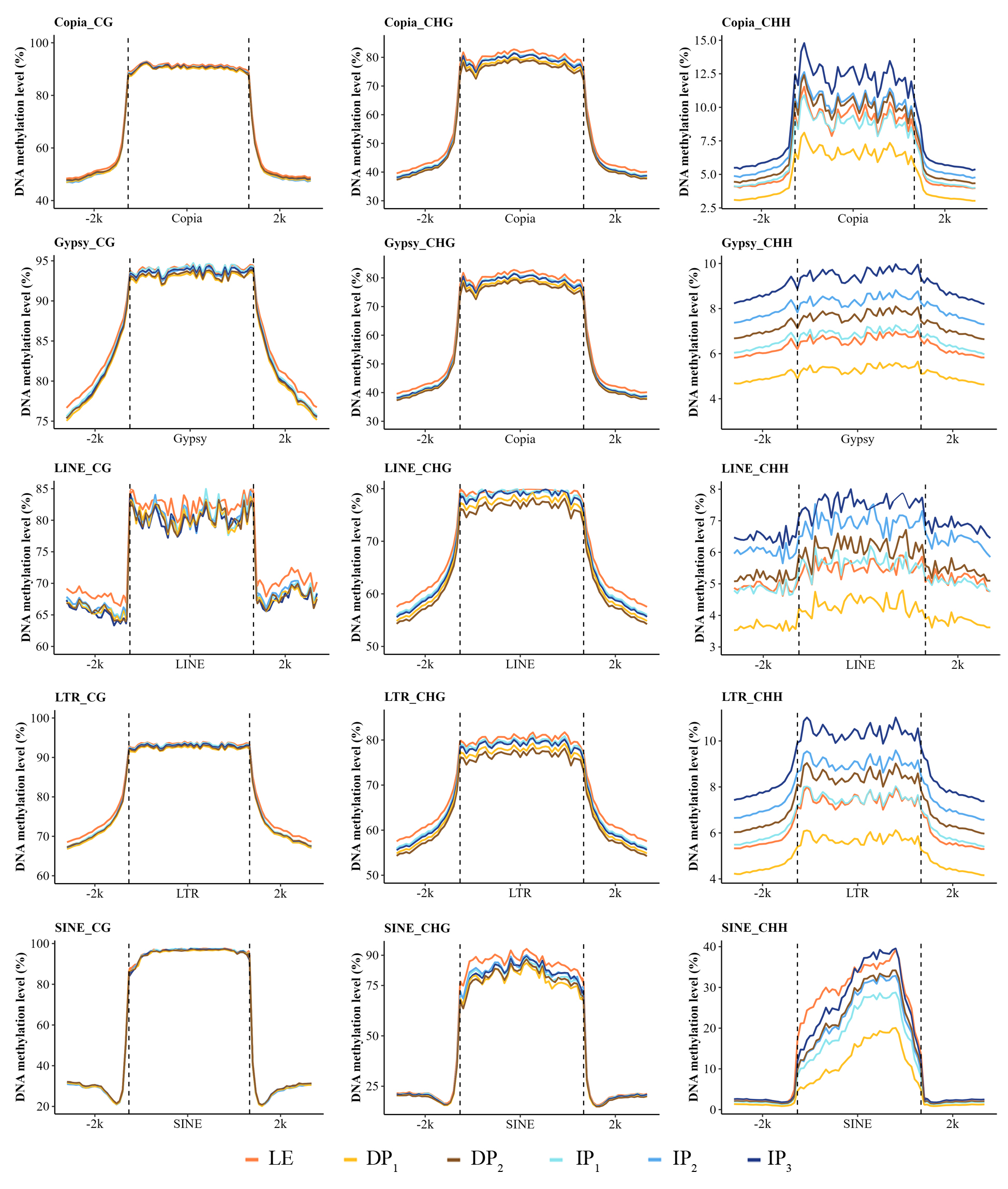


**Figure S2.** Characteristics of DNA methylation patterns in different types of transposable elements.


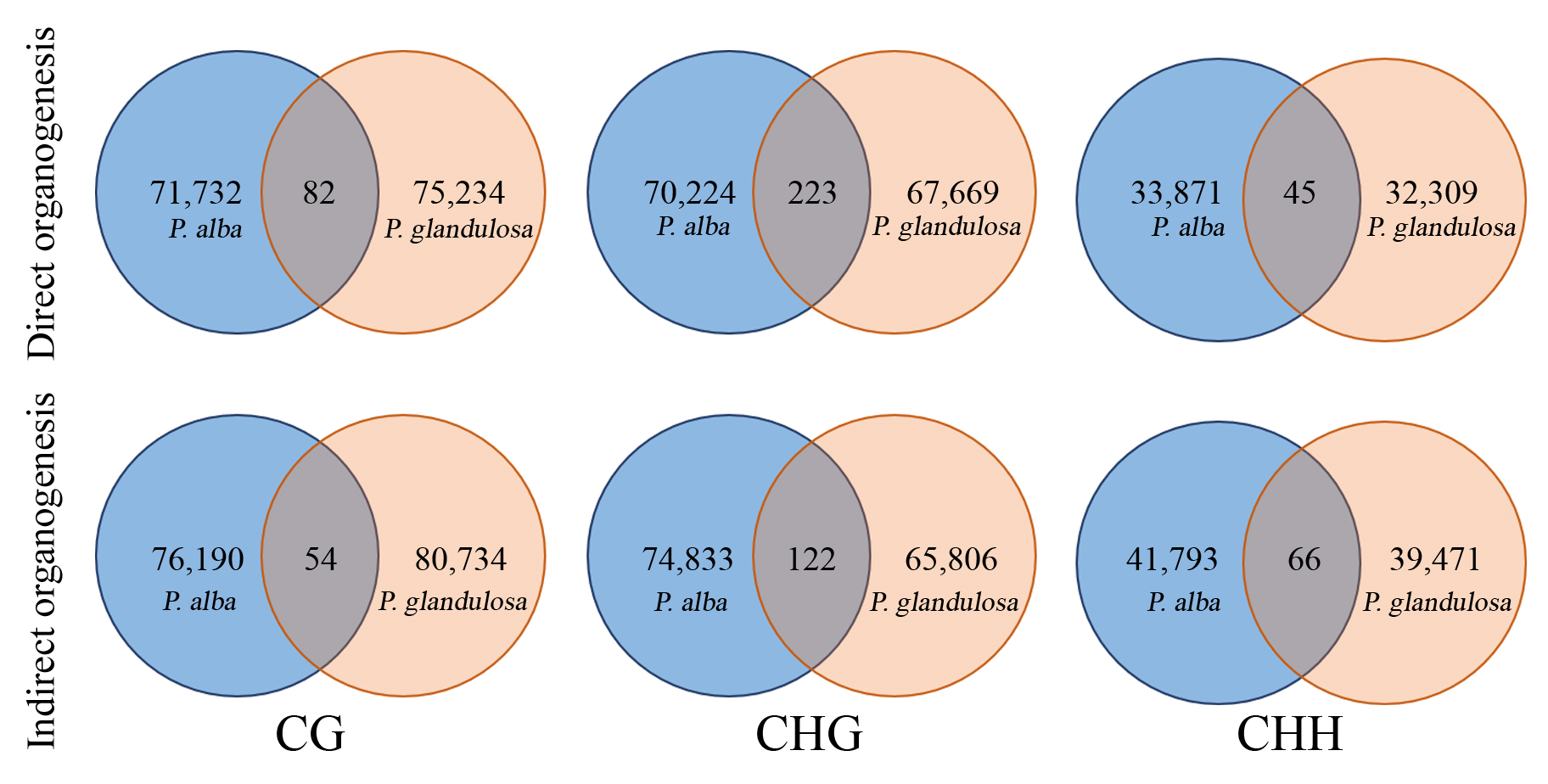


**Figure S3.** Venn diagram counting the numbers of allele-specific DNA methylation regions in CG, CHG, and CHH contexts.
